# Body Mass Index Trajectory–Specific Changes in Economic Circumstances: A Person-Oriented Approach Among Midlife and Ageing Finns

**DOI:** 10.3390/ijerph17103668

**Published:** 2020-05-22

**Authors:** Jatta Salmela, Tea Lallukka, Elina Mauramo, Ossi Rahkonen, Noora Kanerva

**Affiliations:** 1 Department of Public Health, University of Helsinki, P.O. Box 20, 00014 Helsinki, Finland; tea.lallukka@helsinki.fi (T.L.); elina.mauramo@helsinki.fi (E.M.); ossi.rahkonen@helsinki.fi (O.R.); noora.kanerva@helsinki.fi (N.K.); 2 Finnish Institute of Occupational Health, P.O. Box 18, 00032 Helsinki, Finland

**Keywords:** body mass index, economic difficulties, household income, obesity, sequence analysis, trajectory modeling, weight gain

## Abstract

Economic disadvantage is related to a higher risk of adulthood obesity, but few studies have considered whether changes in economic circumstances depend on a person’s body mass index (BMI) trajectory. We identified latent BMI trajectories among midlife and ageing Finns and captured individual-level changes in economic circumstances within the BMI trajectories utilizing sequence analysis. We used the Helsinki Health Study cohort data of initially 40–60-year-old Finnish municipal employees, with four survey questionnaire phases (2000–2017). Each survey included identical questions on height and weight, and on economic circumstances incorporating household income and current economic difficulties. Based on computed BMI, we identified participants’ (*n* = 7105; 82% women) BMI trajectories over the follow-up using group-based trajectory modeling. Four BMI trajectories were identified: stable healthy weight (34% of the participants), stable overweight (42%), overweight to class I obesity (20%), and stable class II obesity (5%). Lower household income level and having economic difficulties became more common and persistent when moving from lower- to higher-level BMI trajectories. Differences in household income widened over the follow-up between the trajectory groups, whereas economic difficulties decreased equally in all trajectory groups over time. Our study provides novel information on the dynamic interplay between long-term BMI changes and economic circumstances.

## 1. Introduction

People with economic disadvantage, such as those having low income or wealth or living in poverty, have an increased risk of unhealthy weight gain and obesity in high-income countries [1,2,3]. Economic disadvantage is assumed to contribute to obesity, for instance, through food insecurity and limited possibilities of exercise activities. However, obesity may also contribute to subsequent lower income, for instance, through discrimination or productivity differences [1,4,5]. Adverse economic circumstances tend to be long-lasting and accumulate over time, even over generations [6], which further increases the risk of developing obesity [7,8]. Given that changes in underlying circumstances can differentially affect the development of chronic diseases [9], more attention should be paid to the dynamic interplay between changes in economic circumstances and changes in body weight. Whereas some aspects of socioeconomic circumstances (e.g., education) are mostly constant, material aspects (e.g., income and economic difficulties) may vary more over time [10,11], which potentially has an impact on changes in health behavior and consequently in body weight.

The associations found between economic disadvantage and obesity are mainly based on studies that have used a static measure of body mass index (BMI)—measured at only one timepoint—as an outcome [4,12], repeated cross-sectional studies [11], or longitudinal studies that assume homogeneity in BMI changes among study populations [13,14]. In recent years, there has been a growing interest in trajectory modeling, which is able to capture distinct subgroups in the developmental patterns of BMI changes over time. Few studies have identified latent BMI trajectories in adulthood and examined the role of economic circumstances, such as income, assets, poverty, and food insecurity, in them [15,16,17]. Although these studies could have considered the changing nature of economic circumstances by treating economic variables as time-varying covariates, they did not capture the variety of individual-level changes in economic circumstances within BMI trajectories.

This study is, to our knowledge, the first that uses a person-oriented approach to describe BMI trajectory-specific and individual-level changes in economic circumstances. We do that by using group-based trajectory modeling together with sequence analysis. Here, we build upon our previous study where we identified four ascending BMI trajectories from early to late adulthood among Finnish municipal employees [18]. Our aim is to demonstrate how changes in adulthood household income and current economic difficulties differ depending on a person’s BMI trajectory, and to provide new insights to capture the heterogeneity in economic circumstances within BMI trajectories.

## 2. Materials and Methods

### 2.1. Study Design and Participants

We used data from the Helsinki Health Study, a large prospective cohort study on social and work-related determinants of health. All the employees of the City of Helsinki in Finland aged 40, 45, 50, 55, and 60 (*n* = 13,346) were invited to the Phase 1 survey (2000–2002), resulting in a response rate of 67% (*n* = 8960) [19]. Of the participants, 80% were women, representing the gender distribution among the City of Helsinki employees and in the public sector in Finland in general. Follow-up surveys among the Phase 1 participants were conducted in 2007 (Phase 2), 2012 (Phase 3), and 2017 (Phase 4) (corresponding response rates 83%, 79%, and 82%). The final data for the analyses in this study consisted of 7105 participants, after excluding participants with missing information on BMI (*n* = 1838) at two or more timepoints, those who were pregnant in Phase 1 (*n* = 20), and a few participants with outliers in BMI values (BMI < 14 kg/m^2^ or BMI > 60 kg/m^2^) (*n* = 3).

The Helsinki Health Study protocol was approved by the ethics committees of the Department of Public Health, University of Helsinki and the health authorities of the City of Helsinki. Appropriate ethical aspects have been followed according to the Helsinki declaration.

### 2.2. Measures

We calculated BMI from self-reported weight and height (BMI in kg/m^2^ units) in all survey phases. When naming the trajectory groups, we defined healthy weight as BMI 18.5–24.9, overweight as BMI 25.0–29.9, class I obesity as BMI 30.0–34.9, and class II obesity as BMI 35.0–39.9, according to the World Health Organization’s BMI classification [20].

Household income from all survey phases was equalized by dividing the typical monthly net income (7–9 levels) by household size that was weighted using the equivalence scale of the Organisation for Economic Co-operation and Development (OECD): the scale equals 1.0 for the respondent, 0.5 for other adults, and 0.3 for children [21]. We divided weighted household income into quartiles, separately for women and men. The final measure included four classes that combined the population of men and women in each survey phase. Current economic difficulties were measured in all survey phases with two questions: “How often do you not have enough money to buy the kind of food or clothing you or your family need?” and “How much difficulty do you have in meeting the payment of bills?”. For both questions, there were five response choices (from always to never and from very little/not at all to very much) indicating the level of difficulties, from which we calculated a sum score with three classes: no (sum score 0), occasional (sum score 1–3) and frequent difficulties (sum score 4–8) [22].

In addition, we included three sociodemographic (gender, age, and marital status) and four socioeconomic variables (education, occupational class, housing tenure, and employment status) into supplementary analyses (Supplementary Table S1). The data for these were derived from the Phase 1 survey, except employment status which was derived from Phase 4 because all participants were employees of the City of Helsinki, Finland, in Phase 1. Marital status was divided into married or cohabiting and others. Education was divided into high (matriculation, college examination or university degree) and low education (vocational school or less). Occupational class was derived from the employer’s registers for those who gave written permission for data linkage (77%) and was completed from the survey questionnaire for the rest. Occupational class was divided into professionals or semi-professionals and routine non-manual employees or manual workers. Housing tenure was divided into owner-occupiers and renters or others. Employment status was classified into current employees, mandatorily retired and disability-retired participants, and others outside employment.

### 2.3. Statistical Analyses

We used group-based trajectory modeling (GBTM) [23] for identifying latent BMI trajectory groups. The number of optimal trajectory groups and trajectory shapes were chosen based on the distinct interpretability of the identified trajectory groups, the existing literature, and the following statistical criteria: Bayesian information criterion (BIC), the average posterior probabilities (APP) of group membership higher than 0.7, and sizes of trajectory groups being at least 5% [23]. Each participant was assigned to the trajectory group for which they had the highest probability of group membership. The APP of group membership was over 0.9 in each trajectory group, and 8% of participants had a posterior probability of group membership lower than 0.7. The model fit statistics are shown in Supplementary Table S2 We ran cross-tabulations with chi-squared tests to describe BMI trajectory groups by economic circumstances in all survey phases (Supplementary Table S3). In addition, cross-tabulations (at one timepoint) were performed for background variables related to economic circumstances to consider their potential role in our main results (Supplementary Table S1).

Sequence analysis was used to capture individual-level changes of economic circumstances within BMI trajectories over the follow-up. Sequence analysis is a descriptive tool whose strength is in visualizing and giving an overall picture of longitudinal data [24,25]. A sequence is an ordered list of elements and episodes, where an element can be a certain status or an event, such as a participant’s income level, at one timepoint. Identical successive elements further constitute episodes, such as a period at a certain income level. In this study, sequence analyses included individual-level descriptive sequence statistics and sequence index plots, performed for all BMI trajectory groups separately. Sequence index plots visualize each participant’s history of economic status over the follow-up, and gather all these individual observations into one plot by utilizing colors [26]. Furthermore, we made status distribution plots as a supplementary analysis to describe the relative proportion of each economic circumstance class in each survey phase. Status distribution plots simplify the overall patterns that are presented in sequence index plots without considering individual-level sequences. We used only complete cases in household income (*n* = 5074) and economic difficulty (*n* = 4925) variables when performing sequence analyses. STATA version 15 (StataCorp LLC, College Station, TX, USA) was used for the analyses.

## 3. Results

### 3.1. Characteristics of the Study Population

Of the final study population, 82% were women (Table 1). Mean BMI was 25.5 (standard deviation, SD, 4.3), and men were slightly more overweight than women. Half of the participants did not have economic difficulties, whereas 10% reported frequent and 36% occasional economic difficulties in Phase 1. Lower household income level became more common during the follow-up among the whole study population, whereas the proportion of participants with economic difficulties decreased over time (Supplementary Table S3).

### 3.2. Body Mass Index Trajectory Groups

**Figure 1:**
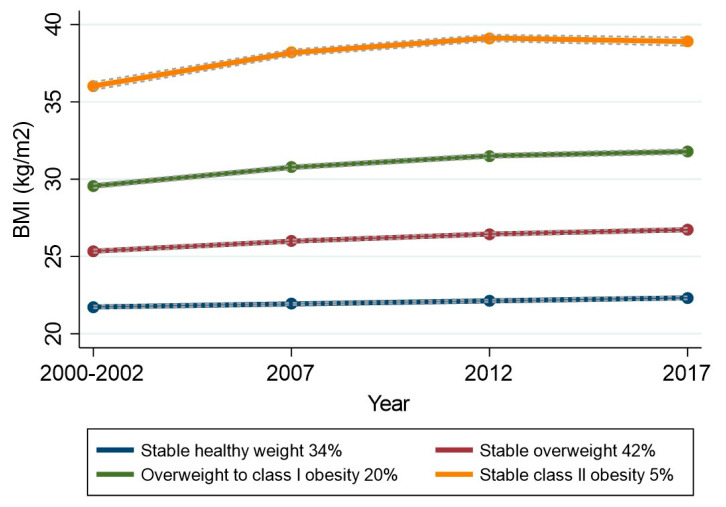
Body mass index (BMI) trajectory groups and their prevalence (%), identified by group-based trajectory modeling (group means and fitted lines with 95% confidence intervals).

Using GBTM, a model with four slightly ascending BMI trajectories was selected—based on the model selection criteria explained in Section 2—among the study population (Figure 1). The groups were named stable healthy weight (34% of participants), stable overweight (42%), overweight to class I obesity (20%) and stable class II obesity (5%) trajectories. Mean weight gain for corresponding trajectory groups from Phase 1 to Phase 4 were 1.2 kg (SD 5.4), 3.5 kg (SD 7.6), 5.7 kg (SD 10.1), and 7.1 kg (SD 13.6). BMI trajectory groups differed by participants’ economic circumstances in all survey phases, adverse economic circumstances being more common in higher-level trajectory groups (Supplementary Table S3).

### 3.3. Sequence Analyses

#### 3.3.1. BMI Trajectories and Household Income

**Figure 2:**
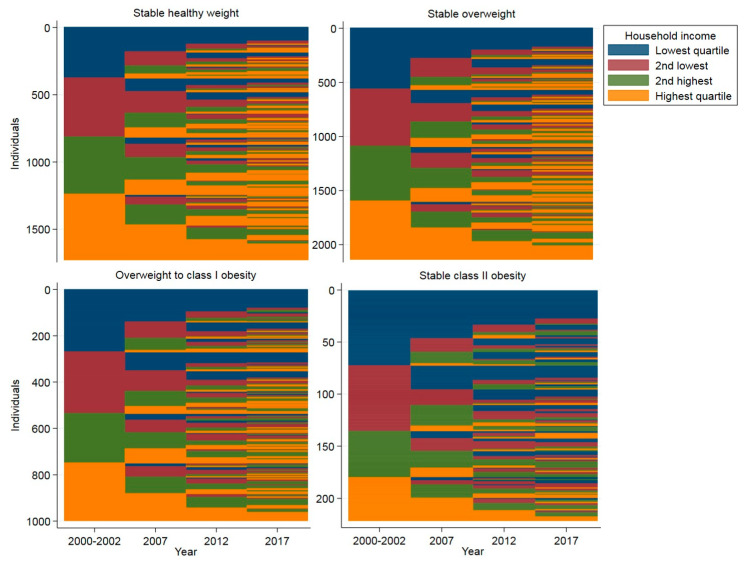
Sequence index plots of household income by the body mass index (BMI) trajectory groups: individual-level sequences in household income quartiles visualized over the follow-up. The y-axis includes all the individual observations within a BMI trajectory group, and the x-axis demonstrates the follow-up time. Each participant’s household income level sequence has been drawn in a horizontal line.

Individual-level changes in household income level over the follow-up, by the BMI trajectory groups, are plotted in Figure 2. Each element—that is, household income quartile—has been visualized with a different color. The y-axis includes all the individual observations within a BMI trajectory group, whereas the x-axis demonstrates the follow-up time (Phases 1–4, years 2000–2017). Thus, each participant’s household income level sequence has been drawn in a horizontal line. Aggregated statistics from these sequences are presented in Table 2. In the stable healthy weight trajectory group, constant belonging to the highest income quartile over the follow-up was the most common sequence, found for 7% of those participants who were assigned to that trajectory group (Table 2). In the other, higher-level BMI trajectory groups, constant belonging to the lowest income quartile was the most common sequence. On average, participants in the higher-level BMI trajectory groups belonged more often in the lowest income quartile during the follow-up, and less often in the highest income quartile, compared to the lower-level BMI trajectory groups (average frequency, Table 2). The average number of different elements and episodes, which indicates the amount of fluctuation and transitions, did not differ between the trajectory groups. The status distribution plots, gathering the individual sequence data together, demonstrate that the differences in household income level widened between the BMI trajectory groups over the follow-up (Supplementary Figure S1).

#### 3.3.2. BMI Trajectories and Current Economic Difficulties

**Figure 3:**
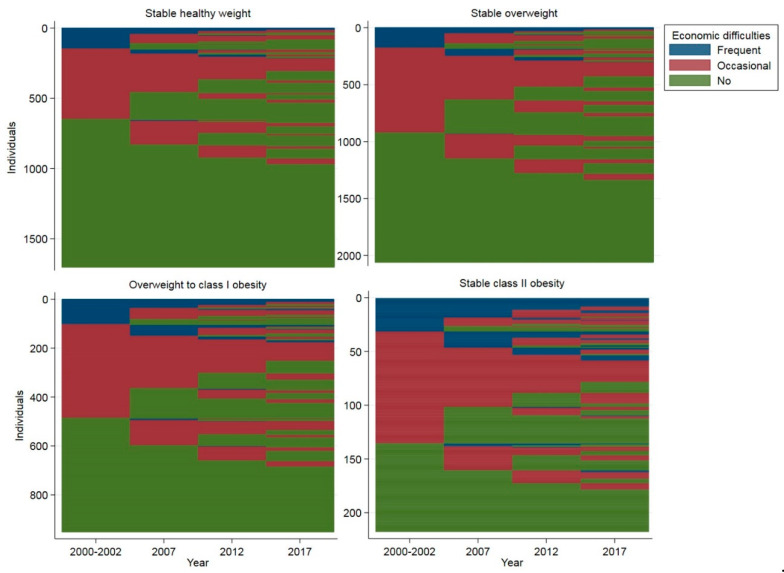
Sequence index plots of household income by the body mass index (BMI) trajectory groups: individual-level sequences in household income quartiles visualized over the follow-up. The y-axis includes all the individual observations within a BMI trajectory group, and the x-axis demonstrates the follow-up time. Each participant’s household income level sequence has been drawn in a horizontal line.

Corresponding sequence index plots for economic difficulties are shown in Figure 3, and aggregated sequence statistics are tabulated in Table 3. Constantly having no economic difficulties over the follow-up was the most common sequence in each BMI trajectory group, with a greater proportion in the lower-level BMI trajectories. For instance, 43% of participants who were assigned to the stable healthy weight trajectory group did not have any economic difficulties during the follow-up, whereas the proportion among those assigned to the stable class II obesity trajectory group was 18%. On average, participants in the lower-level BMI trajectory groups did not experience economic difficulties most of the time (average frequency, Table 3). When moving toward higher-level BMI trajectory groups, time without economic difficulties shortened (average frequency). Participants in the higher-level BMI trajectory groups had more fluctuations in the amount of economic difficulties (average number of different elements) and transitions between economic difficulty classes (average number of episodes) during the follow-up. The relative proportions of having economic difficulties decreased in all BMI trajectory groups over time (Supplementary Figure S2).

## 4. Discussion

We identified latent BMI trajectories among midlife and ageing Finns and examined whether long-term economic circumstances and changes in them differ between the BMI trajectory groups during a 17-year follow-up. The identified BMI trajectory groups were stable healthy weight, stable overweight, overweight to class I obesity and stable class II obesity. Having a lower income level and occasional or frequent economic difficulties was constantly more common in the higher-level BMI trajectory groups, and the differences in household income level widened between the trajectory groups over time. Participants who were assigned to the higher-level BMI trajectory groups remained in the lowest income level for a longer time and without current economic difficulties a shorter time. They also had more fluctuation in having economic difficulties.

Our results support the cross-sectional findings of the prevalence and trends of overweight and obesity among the general adult population in Finland [27]. Additionally, the results are consistent with previous studies that identified latent BMI trajectories among adult populations, typically yielding four to five ascending trajectories [16,28,29,30]. Since obesity is associated with several negative health outcomes [31,32,33], people who are vulnerable to unhealthy weight gain are also exposed to those outcomes. Living with low income and food insecurity have been associated with rising BMI trajectory levels among young to middle-aged Canadians [15]. Additionally, a US study found that living in poverty was associated with a rising BMI trajectory level among adults, but the association disappeared after adjusting for sociodemographic and socioeconomic variables [16]. Our study contributes to the existing research by focusing more on the fluctuating nature of economic circumstances and demonstrating that these fluctuations differ depending on participants’ BMI trajectories.

Participants who were assigned to the trajectories of developing obesity experienced not only more economic difficulties but also more changes in the extent of economic difficulties over time. Although the amount of transitions in household income did not differ between the BMI trajectory groups, the status distribution plots revealed that the transitions were more likely negative in the higher-level BMI trajectory groups. Economic insecurity can lead to instability in health and health behavior, and it has also been associated with weight gain [34,35]. On the other hand, despite stress and other negative health consequences that result from transient and repetitive financial hardship, prolonged economic disadvantage can be even more detrimental for health [36]. Financial stress is often part of a larger proliferation of stressors related to family, job, and environmental factors, which occur more commonly among people with disadvantageous social and economic status. Instead, people with higher income are more likely engaged in healthy behaviors [37], which may result from having enough money to buy healthy foods and have more exercise activities, and from cultural acceptability in neighborhoods and workplaces to make healthy choices.

Although we were able to capture often unrecognized individual-level changes in economic circumstances by using sequence analysis, the method does not enable us to explain whether other factors explain the differences in changes of economic circumstances between BMI trajectories. In previous studies, associations between household income and obesity seem to be explained by other socioeconomic measures, especially by education and occupational class [11,38,39,40]. On the other hand, it may also be the other way around [41]. Economic difficulties, however, reflect not only material circumstances, but also other aspects of economic disadvantage that are associated with obesity, such as mental challenges [42], financial and psychosocial stress [43,44] and food insecurity [45]. Psychosocial and cultural factors are argued to play an even more important role in the development of obesity than material resources [37]. Overall, economic difficulties seem to be independently associated with weight gain and obesity, whereas household income is more tightly related to other socioeconomic factors. In our study, participants with higher-level BMI trajectories were more likely to be lower educated, of lower occupational class, and to live in rented housing in Phase 1 (Supplementary Table S1). In addition, they were more likely to be unemployed or retired due to disability at the end of the follow-up, which can partly explain the widening differences in household income between the BMI trajectory groups.

The widening economic disparity in BMI trajectories indicates a cumulative, disadvantageous interplay between economic circumstances and unhealthy weight gain. Persistent economic hardship increases the risk of reduced physical, psychological and cognitive functioning [46], and that can further increase the risk of unhealthy weight gain. Several studies have demonstrated the accumulation of adverse socioeconomic circumstances to be related to adulthood obesity, especially among women [47,48,49]. As in other chronic diseases, inequalities in the development of obesity often begin during early childhood [9], since several childhood social and economic circumstances are associated with adulthood obesity [4,18]. Even grandparents’ chronic poverty has been associated with grandchildren’s unfavorable weight gain [6], indicating that the cumulative effects of economic disadvantage on obesity can even reach over generations, for instance, through environmental, socio-psychological, and biological mediation. Our previous study [18] showed that most of the adulthood weight gain emerged during young adulthood, which emphasizes the importance of early recognition of and intervening on the risk factors of unhealthy weight gain.

This study has some limitations. First, the cohort is not representative of the Finnish general population because it does not include people from the private sector or outside the labor market, and most of the participants are women. Second, the data on BMI and economic circumstances are derived from self-reports which may be biased, e.g., the calculated BMIs are probably rather underestimates than overestimates [50], whereas negative affectivity among participants with higher BMI may have resulted overestimates in negative responses in economic difficulty measure [51,52]. Third, the identified BMI trajectory groups are simplifications of the real individual-level BMI trajectories [23], and the choice of method and model selection may affect the shape and number of the trajectories [53]. Likewise, changes in economic circumstances are approximations because the participants may have had additional transitions between the four observed timepoints. Last, missing information in the variables may affect the results. However, the identified BMI trajectories well illustrated the situation among the study population after several sensitivity analyses (such as complete case analysis, drop-out analysis, and consideration of cross-sectional BMI distributions). Also, participants with complete data on economic circumstances had only a slightly lower mean BMI in each phase compared to the whole study population. Altogether, the differences in economic circumstances between the BMI trajectory groups are probably slightly greater and widen more over time than our results show.

The main strength of this study is the large, prospective cohort dataset with identical questionnaires at four timepoints. The participants, being a majority of women, represent the target population well––midlife and ageing Finns with public sector employment backgrounds [19,54]––and the results can be generalized to that population. Previous analysis showed that non-response does not seriously bias analyses of social class inequalities in health in our data [55]. Occupational class and income differences between non-responders and participants in Phase 1 were relatively minor and inconsistent [19,54]. Our comprehensive dataset enabled us to use more sophisticated, person-oriented methods to examine long-term changes in participants’ BMI with combined information on current economic circumstances. The uniqueness of this study is in the consideration of population heterogeneity in the changes of BMI and economic circumstances at the same time, which has not been previously done. Performing sequence analyses for economic circumstances within the BMI trajectory groups is a step further in trying to understand how varying economic circumstances are associated with weight changes in the long-term.

## 5. Conclusions

This person-oriented cohort study showed that prolonged and volatile economic disadvantage was more likely to occur among midlife and ageing Finns with developing obesity. Differences in household income widened between the BMI trajectory groups during the 17-year follow-up, which emphasizes the importance of early intervention among economically disadvantaged people. Our study contributes to the existing literature by capturing population heterogeneity in long-term changes of economic circumstances, and by demonstrating how these changes differ depending on a person’s BMI trajectory. Future studies should take into consideration the changing nature of economic circumstances when examining the mechanisms that explain the role of economic circumstances in long-term, unhealthy weight gain. In addition, stressors linked with financial hardship and weight gain should be recognized when planning and targeting obesity prevention acts.

## Figures and Tables

**Table 1:** Characteristics of the study population among women and men in Phase 1 (2000–2002).

	Total, *n* (%)	Women, *n* (%)	Men, *n* (%)
***N***	7105	5790 (82)	1315 (19)
**Age**			
40	1337 (19)	1123 (19)	214 (16)
45	1475 (21)	1238 (21)	237 (18)
50	1571 (22)	1295 (22)	276 (21)
55	1849 (26)	1462 (25)	387 (29)
60	873 (12)	672 (12)	201 (15)
**BMI ^1^, mean (SD ^2^)**	25.5 (4.3)	25.3 (4.3)	26.3 (3.9)
**Household income**			
Highest quartile	1706 (24)	1385 (24)	321 (24)
2^nd^ highest	1601 (23)	1284 (22)	317 (24)
2^nd^ lowest	1813 (26)	1482 (26)	331 (25)
Lowest quartile	1807 (25)	1473 (25)	334 (25)
**Economic difficulties**			
No	3723 (52)	3022 (52)	701 (53)
Occasional	2574 (36)	2094 (36)	480 (37)
Frequent	731 (10)	606 (11)	125 (10)

^1^ BMI = body mass index. ^2^ SD = standard deviation.

**Table 2:** Characteristics of sequences in household income by the body mass index (BMI) trajectory groups over the follow-up (2000–2017).

	BMI Trajectory Group				
Household Income, *n* = 5074	Stable Healthy Weight, *n* = 1724	Stable Overweight, *n* = 2132	Overweight to Class I Obesity, *n* = 997	Stable Class II Obesity, *n* = 221	Min/Max
**5 most common** **sequences (%) ^1^**	4444 (6.6) 1111 (5.9) 4344 (2.7) 3444 (2.6) 2211 (2.6)	1111 (8.3) 4444 (5.6) 1211 (3.1) 4433 (2.8) 2111 (2.7)	1111 (8.0) 2111 (4.8) 4444 (3.5) 1211 (3.1) 2211 (3.1)	1111 (12) 2111 (5.4) 2211 (3.2) 1211 (2.7) 1112/1121 /1311 (2.3)	
**Average frequency ^2^ (SD ^3^) in:**					
Highest quartile	1.10 (1.27)	0.90 (1.18)	0.79 (1.10)	0.61 (0.97)	0/4
2^nd^ highest	1.02 (1.01)	0.97 (0.99)	0.97 (1.00)	0.91 (0.99)	0/4
2^nd^ lowest	0.94 (1.00)	0.99 (1.00)	1.02 (0.97)	0.93 (0.95)	0/4
Lowest quartile	0.95 (1.25)	1.14 (1.33)	1.22 (1.35)	1.55 (1.43)	0/4
**Average number of different elements ^4^ (SD) in a sequence**	2.18 (0.70)	2.20 (0.73)	2.22 (0.69)	2.19 (0.76)	1/4
**Average number of episodes ^5^ (SD) in a sequence**	2.54 (0.93)	2.57 (0.96)	2.58 (0.94)	2.54 (0.95)	1/4

^1^ Sequences represented as ordered lists of household income levels (i.e., income quartiles, numbered as 1–4) in Phases 1–4: 1 = lowest income quartile, 2 = 2^nd^ lowest income quartile, 3 = 2^nd^ highest income quartile, 4 = highest income quartile. ^2^ Mean number of the time points over the four study phases, where participants belonged to a certain household income quartile (e.g., belonging to the highest income quartile in two out of four phases). ^3^ SD = standard deviation. ^4^ Element is a certain status in an individual’s sequence (e.g., belonging to the highest income quartile). ^5^ Episode is a constitution of identical successive elements (e.g., belonging to the highest income quartile in Phases 1–3).

**Table 3:** Characteristics of sequences in current economic difficulties by the body mass index (BMI) trajectory groups over the follow-up (2000–2017).

	BMI Trajectory Group				
Economic Difficulties *n* = 4925	Stable Healthy Weight, *n* = 1701	Stable Overweight, *n* = 2057	Overweight to Class I Obesity, *n* = 950	Stable Class II Obesity, *n* = 217	Min/Max
**5 most common** **sequences (%) ^1^**	3333 (43) 2333 (6.8) 2222 (5.3) 2233 (3.9) 3233 (3.8)	3333 (35) 2333 (6.9) 2222 (6.1) 2223 (4.3) 3233 (4.2)	3333 (28) 2222 (7.9) 2333 (6.4) 2223 (5.2) 3323 (4.1)	3333 (18) 2333 (11) 2222 (9.2) 2223/2232 (4.6) 3233 (4.2)	
**Average frequency ^2^ (SD ^3^) in:**					
No difficulties	2.68 (1.44)	2.44 (1.47)	2.15 (1.51)	1.82 (1.50)	0/4
Occasional difficulties	1.08 (1.24)	1.28 (1.27)	1.46 (1.32)	1.60 (1.29)	0/4
Frequent difficulties	0.24 (0.67)	0.28 (0.71)	0.38 (0.84)	0.58 (1.06)	0/4
**Average number of different elements ^4^ (SD) in a sequence**	1.55 (0.58)	1.65 (0.60)	1.70 (0.60)	1.76 (0.57)	1/3
**Average number of episodes ^5^ (SD) in a sequence**	1.81 (0.92)	1.97 (0.98)	2.03 (0.96)	2.14 (0.97)	1/4

^1^ Sequences represented as ordered lists of economic difficulty classes (numbered as 1–3) in Phases 1–4: 1 = frequent economic difficulties, 2 = occasional economic difficulties, 3 = no economic difficulties. ^2^ Mean number of the time points over the four study phases, where participants reported a certain economic difficulty class (e.g., no difficulties in two out of four phases). ^3^ SD = standard deviation. ^4^ Element is a certain status in an individual’s sequence (e.g., belonging to the “no difficulties” class). ^5^ Episode is a constitution of identical successive elements (e.g., belonging to the “no difficulties” class in Phases 1–3).

## Supplementary Materials

The following are available online at https://www.mdpi.com/1660-4601/17/10/3668/s1, Table S1: Cross-tabulations for background variables by the body mass index (BMI) trajectory groups in Phase 1 (2000–2002), Table S2: Model selection of optimal number and shapes of body mass index trajectory groups. Bayesian information criterion (BIC) and the group sizes (%) shown, optimal model bolded, Table S3: Cross-tabulations for household income and current economic difficulties by the body mass index (BMI) trajectory groups among the whole study population. Each of the four survey phases (2000–2017) shown with *p*-value from the chi-squared test, Figure S1: State distribution plots visualizing the relative proportion of each household income quartile by the body mass index trajectory groups over the follow-up (2000–2017), Figure S2: State distribution plots visualizing the relative proportion of each economic difficulty class by the body mass index trajectory groups over the follow-up (2000–2017).

## Abbreviations

APP	Average Posterior Probabilities
BIC	Bayesian Information Criterion
BMI	Body Mass Index
GBTM	Group-Based Trajectory Modeling
OECD	Organisation for Economic Co-operation and Development
SD	Standard Deviation

## References

[1] Finkelstein: E.A., Ruhm C.J., Kosa K.M. (2005). Economic causes and consequences of obesity. Annu Rev. Public Health..

[2] Herzog B., Lacruz M.E., Haerting J., Hartwig S., Tiller D., Medenwald D., Vogt S., Thorand B., Holle R., Bachlechner U. (2016). Socioeconomic status and anthropometric changes—A meta-analytic approach from seven German cohorts. Obesity (Silver Spring).

[3] Wolfe J.D., Baker E.H., Scarinci I.C. (2019). Wealth and Obesity Among US Adults Entering Midlife. Obesity (Silver Spring).

[4] Hernandez D.C., Pressler E. (2014). Accumulation of childhood poverty on young adult overweight or obese status: Race/ethnicity and gender disparities. J. Epidemiol. Community Health.

[5] Kim T.J., von dem Knesebeck O. (2018). Income and obesity: What is the direction of the relationship? A systematic review and meta-analysis. BMJ Open.

[6] Li M. (2015). Chronic Exposure of Grandparents to Poverty and Body Mass Index Trajectories of Grandchildren: A Prospective Intergenerational Study. Am. J. Epidemiol..

[7] Lee H., Harris K.M., Gordon-Larsen P. (2009). Life Course Perspectives on the Links Between Poverty and Obesity During the Transition to Young Adulthood. Popul. Res. Policy Rev..

[8] Hiilamo A., Lallukka T., Mänty M., Kouvonen A. (2017). Obesity and socioeconomic disadvantage in midlife female public sector employees: A cohort study. BMC Public Health.

[9] Lynch J., Davey Smith G. (2005). A life course approach to chronic disease epidemiology. Annu. Rev. Public Health.

[10] Zhang Q., Wang Y. (2004). Trends in the Association between Obesity and Socioeconomic Status in U.S. Adults: 1971 to 2000. Obes. Res..

[11] Zhu J., Coombs N., Stamatakis E. (2015). Temporal trends in socioeconomic inequalities in obesity prevalence among economically-active working-age adults in Scotland between 1995 and 2011: A population-based repeated cross-sectional study. BMJ Open.

[12] Conklin A.I., Forouhi N.G., Suhrcke M., Surtees P., Wareham N.J., Monsivais P. (2013). Socioeconomic status, financial hardship and measured obesity in older adults: A cross-sectional study of the EPIC-Norfolk cohort. BMC Public Health.

[13] Botoseneanu A., Liang J. (2011). Social stratification of body weight trajectory in middle-age and older Americans: Results from a 14-year longitudinal study. J. Aging Health.

[14] Insaf T.Z., Shaw B.A., Yucel R.M., Chasan-Taber L., Strogatza D.S. (2014). Lifecourse socioeconomic position and 16 year body mass index trajectories: Differences by race and sex. Prev. Med..

[15] Wang M., Yi Y., Roebothan B., Colbourne J., Maddalena V., Wang P.P., Sun G. (2015). Trajectories of Body Mass Index from Young Adulthood to Middle Age among Canadian Men and Women. Adv. Epidemiol..

[16] Østbye T., Malhotra R., Landerman L.R. (2011). Body mass trajectories through adulthood: Results from the National Longitudinal Survey of Youth 1979 Cohort (1981–2006). Int. J. Epidemiol..

[17] Botoseneanu A., Liang J. (2013). Latent heterogeneity in long-term trajectories of body mass index in older adults. J. Aging Health.

[18] Salmela J., Mauramo E., Lallukka T., Rahkonen O., Kanerva N. (2019). Associations between Childhood Disadvantage and Adult Body Mass Index Trajectories: A Follow-Up Study among Midlife Finnish Municipal Employees. Obes. Facts.

[19] Lahelma E., Aittomäki A., Laaksonen M., Lallukka T., Martikainen P., Piha K., Rahkonen O., Saastamoinen P. (2013). Cohort Profile: The Helsinki Health Study. Int. J. Epidemiol..

[20] World Health Organization Regional Office for Europe. http://www.euro.who.int/en/health-topics/disease-prevention/nutrition/a-healthy-lifestyle/body-mass-index-bmi.

[21] Hagenaars A.J.M., de Vos K., Zaidi M.A. (1994). Poverty Statistics in the Late 1980s: Research Based on Micro-data.

[22] Pearlin L.I., Schooler C. (1978). The structure of coping. J. Health Soc. Behav..

[23] Nagin D.S., Odgers C.L. (2010). Group-based trajectory modeling in clinical research. Annu Rev. Clin. Psychol..

[24] Abbott A. (1995). Sequence Analysis: New Methods for Old Ideas. Annu Rev. Sociol..

[25] Brzinsky-Fay C., Kohler U., Luniak U. (2006). Sequence analysis with Stata. Stata J..

[26] Brzinsky-Fay C., Blanchard P., Bühlmann F., Gauthier J. (2014). Graphical representation of transitions and sequences. Advances in sequence analysis: Theory, method applications.

[27] Lundqvist A., Männistö S., Jousilahti P., Kaartinen N., Mäki P., Borodulin K. (2018). Lihavuus. Terveys, toimintakyky ja hyvinvointi Suomessa-Finterveys 2017 -tutkimus [Health, Functional Capacity and Welfare in Finland-FinHealth 2017 Study].

[28] Wang M., Yi Y., Roebothan B., Colbourne J., Maddalena V., Wang P.P., Sun G. (2016). Body Mass Index Trajectories among Middle-Aged and Elderly Canadians and Associated Health Outcomes. J. Environ. Public Health.

[29] Kelly S.P., Lennon H., Sperrin M., Matthews C., Freedman N.D., Albanes D., Leitzmann M.F., Renehan A.G., Cook M.B. (2018). Body mass index trajectories across adulthood and smoking in relation to prostate cancer risks: The NIH-AARP Diet and Health Study. Int. J. Epidemiol..

[30] De Rubeis V., Cotterchio M., Smith B.T., Griffith L.E., Borgida A., Gallinger S., Cleary S., Anderson L.N. (2019). Trajectories of body mass index, from adolescence to older adulthood, and pancreatic cancer risk; a population-based case-control study in Ontario, Canada. Cancer Causes Control.

[31] Abdelaal M., le Roux C.W., Docherty N.G. (2017). Morbidity and mortality associated with obesity. Ann. Trans. Med..

[32] Nyberg S.T., Batty G.D., Pentti J., Virtanen M., Alfredsson L., Fransson E.I., Goldberg M., Heikkilä K., Jokela M., Knutsson A. (2018). Obesity and loss of disease-free years owing to major non-communicable diseases: A multicohort study. Lancet Public Health.

[33] Taylor V.H., Forhan M., Vigod S.N., McIntyre R.S., Morrison K.M. (2013). The impact of obesity on quality of life. Best Pract. Res. Clin. Endocrinol. Metab..

[34] Watson B. (2018). Does Economic Insecurity Cause Weight Gain Among Canadian Labor Force Participants?. Rev. Income Wealth.

[35] Monsivais P., Martin A., Suhrcke M., Forouhi N.G., Wareham N.J. (2015). Job-loss and weight gain in British adults: Evidence from two longitudinal studies. Soc. Sci. Med..

[36] Pearlin L.I., Schieman S., Fazio E.M., Meersman S.C. (2005). Stress, Health, and the Life Course: Some Conceptual Perspectives. J. Health Soc. Behav..

[37] Godley J., McLaren L. (2010). Socioeconomic status and body mass index in Canada: Exploring measures and mechanisms. Can. Rev. Sociol..

[38] Laaksonen M., Sarlio-Lähteenkorva S., Lahelma E. (2004). Multiple dimensions of socioeconomic position and obesity among employees: The Helsinki Health Study. Obes. Res..

[39] Loman T., Lallukka T., Laaksonen M., Rahkonen O., Lahelma E. (2013). Multiple socioeconomic determinants of weight gain: The Helsinki Health Study. BMC Public Health.

[40] Groth M.V., Fagt S., Stockmarr A., Matthiessen J., Biltoft-Jensen A. (2009). Dimensions of socioeconomic position related to body mass index and obesity among Danish women and men. Scand J. Public Health.

[41] Laaksonen M., Roos E., Rahkonen O., Martikainen P., Lahelma E. (2005). Influence of material and behavioural factors on occupational class differences in health. J. Epidemiol. Community Health.

[42] Laaksonen E., Martikainen P., Lahelma E., Lallukka T., Rahkonen O., Head J., Marmot M. (2007). Socioeconomic circumstances and common mental disorders among Finnish and British public sector employees: Evidence from the Helsinki Health Study and the Whitehall II Study. Int. J. Epidemiol..

[43] Siahpush M., Huang T.T., Sikora A., Tibbits M., Shaikh R.A., Singh G.K. (2014). Prolonged financial stress predicts subsequent obesity: Results from a prospective study of an Australian national sample. Obesity (Silver Spring).

[44] Block J.P., He Y., Zaslavsky A.M., Ding L., Ayanian J.Z. (2009). Psychosocial Stress and Change in Weight Among US Adults. Am. J. Epidemiol..

[45] Moradi S., Mirzababaei A., Dadfarma A., Rezaei S., Mohammadi H., Jannat B., Mirzaei K. (2019). Food insecurity and adult weight abnormality risk: A systematic review and meta-analysis. Eur. J. Nutr..

[46] Lynch J.W., Kaplan G.A., Shema S.J. (1997). Cumulative impact of sustained economic hardship on physical, cognitive, psychological, and social functioning. N. Engl. J. Med..

[47] Giskes K., van Lenthe F.J., Turrell G., Kamphuis C.B., Brug J., Mackenbach J.P. (2008). Socioeconomic position at different stages of the life course and its influence on body weight and weight gain in adulthood: A longitudinal study with 13-year follow-up. Obesity (Silver Spring).

[48] Heraclides A., Brunner E. (2010). Social mobility and social accumulation across the life course in relation to adult overweight and obesity: The Whitehall II study. J. Epidemiol. Community Health.

[49] Gustafsson P.E., Persson M., Hammarström A. (2012). Socio-economic disadvantage and body mass over the life course in women and men: Results from the Northern Swedish Cohort. Eur. J. Public Health.

[50] Connor Gorber S., Tremblay M., Moher D., Gorber B. (2007). A comparison of direct vs. self-report measures for assessing height, weight and body mass index: A systematic review. Obes. Rev..

[51] Laaksonen E., Martikainen P., Lallukka T., Lahelma E., Ferrie J., Rahkonen O., Marmot M., Head J. (2009). Economic difficulties and common mental disorders among Finnish and British white-collar employees: The contribution of social and behavioural factors. J. Epidemiol. Community Health.

[52] Watson D. (1988). Intraindividual and interindividual analyses of positive and negative affect: Their relation to health complaints, perceived stress, and daily activities. J. Pers. Soc. Psychol..

[53] Warren J.R., Luo L., Halpern-Manners A., Raymo J.M., Palloni A. (2015). Do Different Methods for Modeling Age-Graded Trajectories Yield Consistent and Valid Results?. AJS.

[54] Laaksonen M., Aittomäki A., Lallukka T., Rahkonen O., Saastamoinen P., Silventoinen K., Lahelma E. (2008). Register-based study among employees shows small non-participation bias in health surveys and check-ups. J. Clin. Epidemiol..

[55] Martikainen P., Laaksonen M., Piha K., Lallukka T. (2007). Does survey bias the association between occupational social class and health?. Scand J. Public Health.

