# Supplementary material for: Body Mass Index Trajectory–Specific Changes in Economic Circumstances: A Person-Oriented Approach Among Midlife and Ageing Finns
Source: Int J Environ Res Public Health. 2020 May 22;17(10):3668. doi: 10.3390/ijerph17103668 (PMC7277894; doi:10.3390/ijerph17103668)
Supplement: Supplementary file 1 [file ijerph-17-03668-s001.zip › ijerph-803737 - supplementary final.docx]

**Table 1.** Cross-tabulations for background variables ^1^ by the body mass index (BMI) trajectory groups in Phase 1 (2000–2002).

|  |  | **BMI Trajectory Group, *n* (%)** | | | | ***p*-value for χ^2^ test** |
| --- | --- | --- | --- | --- | --- | --- |
|  | **Total, *n* (%)** | **Stable Healthy Weight** | **Stable Overweight** | **Overweight to Class I Obesity** | **Stable Class II Obesity** |  |
| **Gender** |  |  |  |  |  | < 0.001 |
| Woman | 5790 (82) | 2076 (87) | 2313 (78) | 1123 (80) | 278 (83) |  |
| Man | 1315 (19) | 315 (13) | 657 (22) | 287 (20) | 56 (17) |  |
| **Age** |  |  |  |  |  | < 0.001 |
| 40 | 1337 (19) | 500 (21) | 542 (18) | 238 (17) | 57 (17) |  |
| 45 | 1475 (21) | 521 (22) | 569 (19) | 310 (22) | 75 (23) |  |
| 50 | 1571 (22) | 541 (23) | 676 (23) | 285 (20) | 69 (21) |  |
| 55 | 1849 (26) | 582 (24) | 768 (26) | 399 (28) | 100 (30) |  |
| 60 | 873 (12) | 247 (10) | 415 (14) | 178 (13) | 33 (10) |  |
| **Marital status** |  |  |  |  |  | < 0.001 |
| Married /  cohabiting | 4976 (70) | 1694 (71) | 2091 (71) | 995 (71) | 196 (59) |  |
| Others | 2089 (30) | 683 (29) | 865 (29) | 406 (29) | 135 (41) |  |
| **Education** |  |  |  |  |  | < 0.001 |
| High | 1897 (27) | 812 (34) | 757 (26) | 279 (20) | 49 (15) |  |
| Low | 5146 (73) | 1556 (66) | 2188 (74) | 1120 (80) | 282 (85) |  |
| **Occupational class** |  |  |  |  |  | < 0.001 |
| Professional /  semi-professional | 3316 (51) | 1277 (59) | 1358 (50) | 572 (45) | 109 (36) |  |
| Routine /  manual worker | 3137 (49) | 905 (42) | 1341 (50) | 698 (55) | 193 (64) |  |
| **Housing tenure** |  |  |  |  |  | < 0.001 |
| Owner-occupier | 4800 (68) | 1746 (74) | 2012 (68) | 883 (63) | 159 (48) |  |
| Renter / others | 2259 (32) | 629 (27) | 939 (32) | 519 (37) | 172 (52) |  |
| **Employment status, Phase 4 ^2^** |  |  |  |  |  | < 0.001 |
| Employed | 2363 (38) | 888 (42) | 948 (36) | 440 (36) | 87 (32) |  |
| Mandatorily retired | 3451 (55) | 1130 (53) | 1509 (58) | 666 (54) | 146 (53) |  |
| Disability-retired | 354 (5.7) | 80 (3.8) | 130 (5.0) | 108 (8.8) | 36 (13) |  |
| Others outside employment | 71 (1.1) | 20 (0.9) | 33 (1.3) | 12 (1.0) | 6 (2.2) |  |

^1^ Data for background variables were derived from the Helsinki Health Study survey questionnaires. ^2^ Employment status was derived from the Phase 4 (2017) survey because all participants were employees of the City of Helsinki, Finland, in Phase 1.

**Table 2.** Model selection of optimal number and shapes of body mass index trajectory groups. Bayesian information criterion (BIC) and the group sizes (%) shown, optimal model bolded.

| **Number**  **of Groups** | **Trajectory Shapes ^1^** | **BIC ^2^**  **(*n* = 26,622)** | **BIC ^3^**  **(*n* = 7105)** | **Group 1 (%)** | **Group 2 (%)** | **Group 3 (%)** | **Group 4 (%)** | **Group 5 (%)** |
| --- | --- | --- | --- | --- | --- | --- | --- | --- |
| 1 | 3 | -78,562 | -78,559 | 100.0 |  |  |  |  |
| 2 | 3 3 | -71,479 | -71,473 | 73.4 | 26.6 |  |  |  |
| 3 | 3 3 3 | -67,626 | -67,612 | 51.6 | 39.3 | 9.1 |  |  |
| 4 | 3 3 3 3 | -65,299 | -65,286 | 33.8 | 41.6 | 19.8 | 4.8 |  |
| 5 | 3 3 3 3 3 | -64,076 | -64,060 | 26.7 | 38.3 | 24.0 | 9.1 | 2.0 ^4^ |
| 4 | 2 3 3 3 | -65,294 | -65,281 | 33.8 | 41.6 | 19.8 | 4.8 |  |
| 4 | 2 2 3 3 | -65,289 | -65,277 | 33.8 | 41.6 | 19.8 | 4.8 |  |
| 4 | 2 2 2 3 | -65,284 | -65,273 | 33.8 | 41.6 | 19.8 | 4.8 |  |
| 4 | 2 2 2 2 | -65,279 | -65,269 | 33.8 | 41.6 | 19.8 | 4.8 |  |
| **4** | **1 2 2 2** | **-65,274** | **-65,264** | **33.8** | **41.6** | **19.8** | **4.8** |  |
| 4 | 1 1 2 2 | -65,279 | -65,270 | 33.8 | 41.6 | 19.9 | 4.8 |  |
| 4 | 1 1 1 2 | -65,304 | -65,296 | 33.7 | 41.6 | 19.9 | 4.8 |  |
| 4 | 1 1 1 1 | -65,346 | -65,338 | 33.8 | 41.6 | 19.8 | 4.8 |  |
| 4 | 1 2 2 2 ^5^ | -68,291 | -68,275 | 33.5 | 41.5 | 20.2 | 4.8 |  |

^1^ Trajectory shapes: 0 = intercept, 1 = linear, 2 = quadratic, 3 = cubic. ^2^ Bayesian information criterion (BIC) at longitudinal level. ^3^ Bayesian information criterion (BIC) in subject level. ^4^ Group size considered too small. ^5^ Dropout model* included. *Haviland AM, Jones BL, Nagin DS. Group-based Trajectory Modeling Extended to Account for Nonrandom Participant Attrition. *Sociol Methods Res.* **2011**, 40, 367–390.

**Table 3.** Cross-tabulations for household income and current economic difficulties by the body mass index (BMI) trajectory groups among the whole study population. Each of the four survey phases (2000–2017) shown with *p*-value from the chi-squared test.

|  |  | **BMI Trajectory Group, *n* (%)** | | | | ***p*-value for** **χ^2^ test** |
| --- | --- | --- | --- | --- | --- | --- |
|  | **Total, *n* (%)** | **Stable Healthy Weight** | **Stable Overweight** | **Overweight to Class I Obesity** | **Stable Class II Obesity** |  |
| **Household income** | | | | | | |
| **Phase 1 (2000-2)** |  |  |  |  |  | < 0.001 |
| Highest quartile | 1706 (25) | 617 (27) | 710 (25) | 322 (23) | 57 (18) |  |
| 2^nd^ highest | 1601 (23) | 563 (24) | 678 (23) | 291 (21) | 69 (21) |  |
| 2^nd^ lowest | 1813 (26) | 606 (26) | 737 (25) | 371 (27) | 99 (31) |  |
| Lowest quartile | 1807 (26) | 531 (23) | 778 (27) | 399 (29) | 99 (31) |  |
| **Phase 2 (2007)** |  |  |  |  |  | < 0.001 |
| Highest quartile | 1497 (23) | 565 (26) | 620 (23) | 264 (20) | 48 (16) |  |
| 2^nd^ highest | 1720 (26) | 597 (27) | 714 (26) | 327 (25) | 82 (27) |  |
| 2^nd^ lowest | 1684 (26) | 562 (26) | 707 (26) | 345 (27) | 70 (23) |  |
| Lowest quartile | 1650 (25) | 476 (22) | 702 (26) | 365 (28) | 107 (35) |  |
| **Phase 3 (2012)** |  |  |  |  |  | < 0.001 |
| Highest quartile | 1333 (21) | 549 (26) | 537 (20) | 208 (16) | 39 (13) |  |
| 2^nd^ highest | 1515 (24) | 542 (25) | 620 (23) | 292 (23) | 61 (20) |  |
| 2^nd^ lowest | 1511 (24) | 468 (22) | 642 (24) | 327 (26) | 74 (24) |  |
| Lowest quartile | 2048 (32) | 595 (28) | 875 (33) | 446 (35) | 132 (43) |  |
| **Phase 4 (2017)** |  |  |  |  |  | < 0.001 |
| Highest quartile | 1363 (22) | 601 (28) | 520 (20) | 207 (17) | 35 (13) |  |
| 2^nd^ highest | 1443 (23) | 474 (22) | 622 (24) | 291 (24) | 56 (20) |  |
| 2^nd^ lowest | 1453 (23) | 485 (23) | 612 (23) | 292 (24) | 64 (23) |  |
| Lowest quartile | 2026 (32) | 575 (27) | 888 (34) | 440 (36) | 123 (44) |  |
| **Economic Difficulties** | | | | | | |
| **Phase 1 (2000-2)** |  |  |  |  |  | < 0.001 |
| No difficulties | 3723 (53) | 1424 (60) | 1532 (52) | 652 (47) | 115 (35) |  |
| Occasional difficulties | 2574 (37) | 744 (31) | 1114 (38) | 565 (41) | 151 (46) |  |
| Frequent difficulties | 731 (10) | 199 (8.4) | 289 (9.8) | 178 (13) | 65 (20) |  |
| **Phase 2 (2007)** |  |  |  |  |  | < 0.001 |
| No difficulties | 3600 (58) | 1345 (64) | 1506 (58) | 633 (51) | 116 (40) |  |
| Occasional difficulties | 2112 (34) | 632 (30) | 888 (34) | 468 (38) | 124 (42) |  |
| Frequent difficulties | 505 (8.1) | 126 (6.0) | 191 (7.4) | 135 (11) | 53 (18) |  |
| **Phase 3 (2012)** |  |  |  |  |  | < 0.001 |
| No difficulties | 3829 (59) | 1452 (67) | 1587 (59) | 659 (52) | 131 (43) |  |
| Occasional difficulties | 2104 (33) | 600 (28) | 901 (33) | 485 (38) | 118 (39) |  |
| Frequent difficulties | 521 (8.1) | 129 (5.9) | 209 (7.7) | 129 (10) | 54 (18) |  |
| **Phase 4 (2017***)* |  |  |  |  |  | < 0.001 |
| No difficulties | 4206 (66) | 1561 (72) | 1786 (67) | 725 (58) | 134 (47) |  |
| Occasional difficulties | 1744 (27) | 507 (23) | 724 (27) | 409 (33) | 104 (37) |  |
| Frequent difficulties | 406 (6.4) | 97 (4.5) | 156 (5.9) | 108 (8.7) | 45 (16) |  |


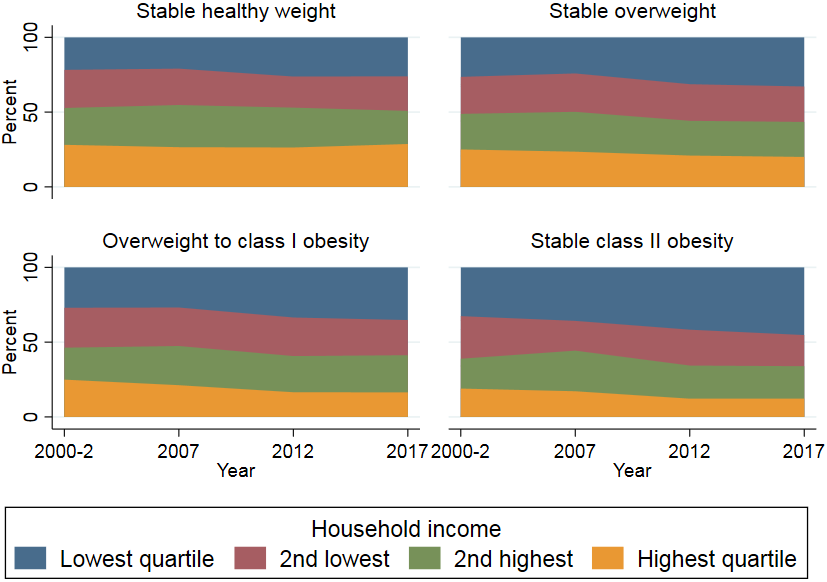


**Figure 1.** State distribution plots visualizing the relative proportion of each household income quartile by the body mass index trajectory groups over the follow-up (2000–2017).


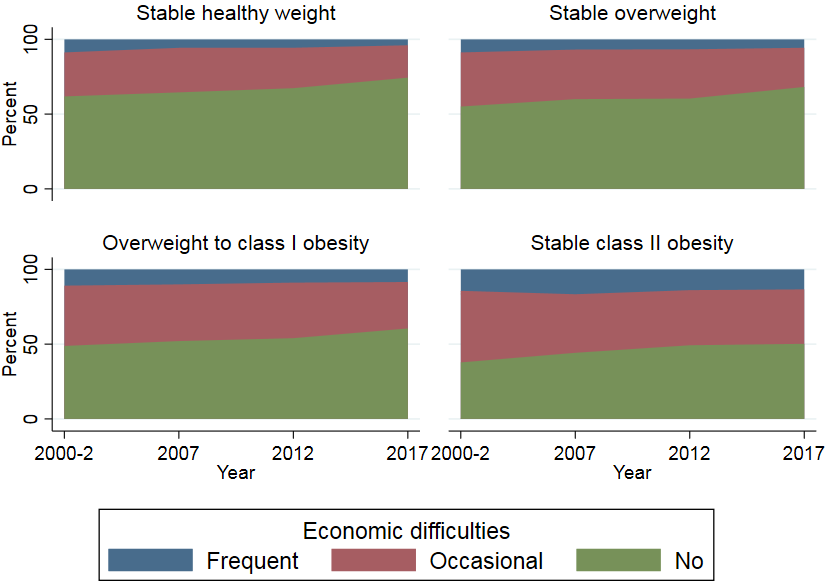


**Figure 2.** State distribution plots visualizing the relative proportion of each economic difficulty class by the body mass index trajectory groups over the follow-up (2000–2017).
