# Supplementary figures and images for: Body Mass Index Trajectory–Specific Changes in Economic Circumstances: A Person-Oriented Approach Among Midlife and Ageing Finns
Source: Int J Environ Res Public Health. 2020 May 22;17(10):3668. doi: 10.3390/ijerph17103668 (PMC7277894; doi:10.3390/ijerph17103668)

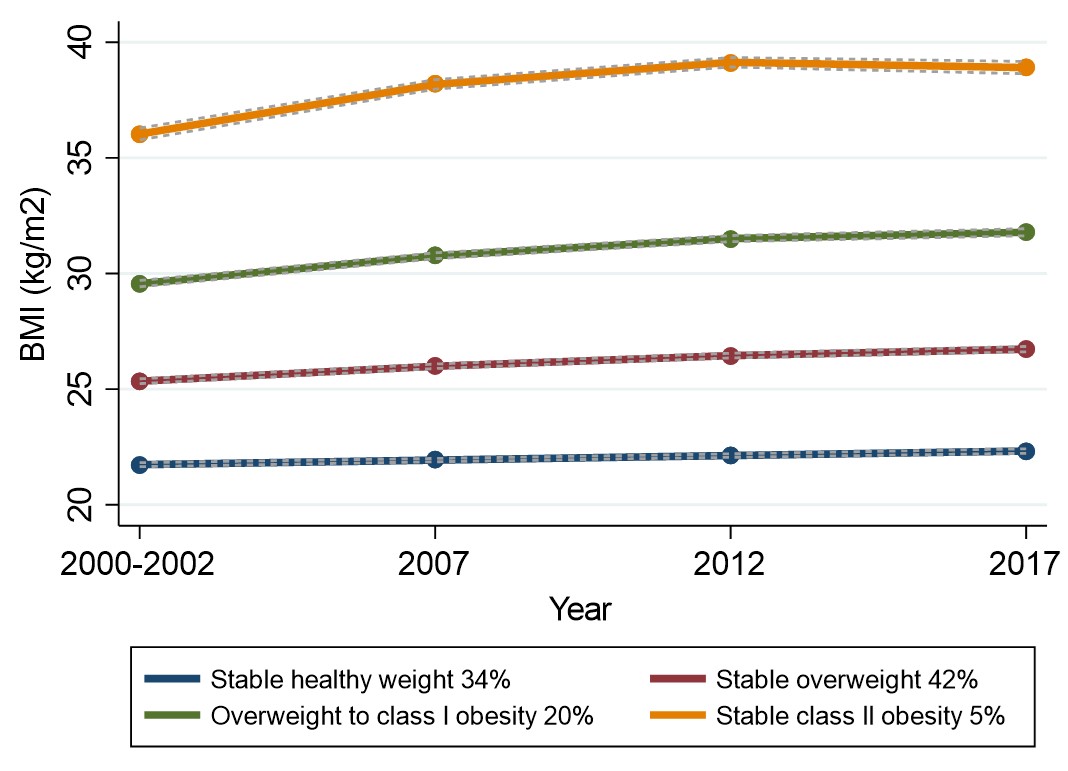

Supplement: Supplementary file 1 [file ijerph-17-03668-s001.zip › Figure1_IJERPH.jpg]

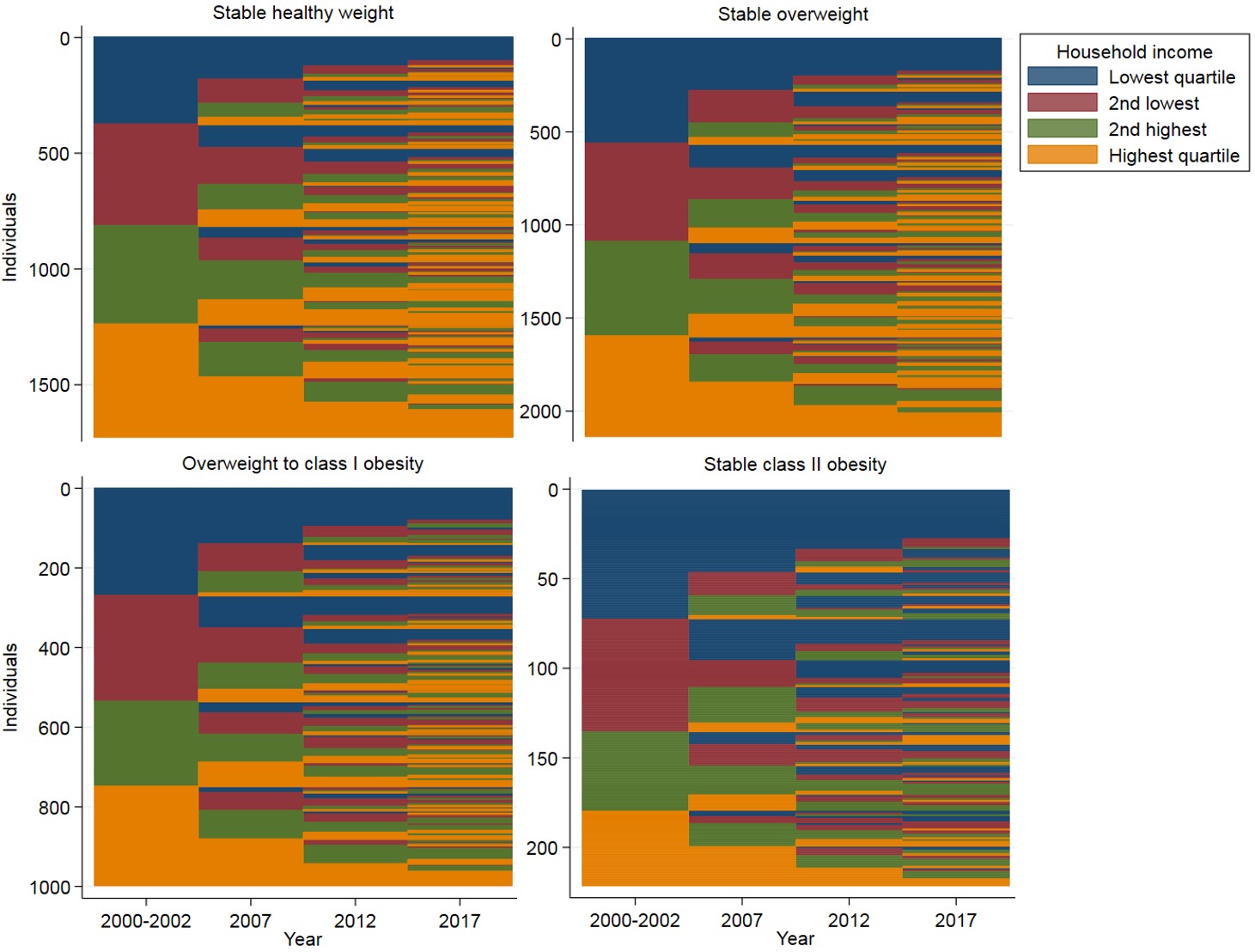

Supplement: Supplementary file 1 [file ijerph-17-03668-s001.zip › Figure2_IJERPH.jpg]

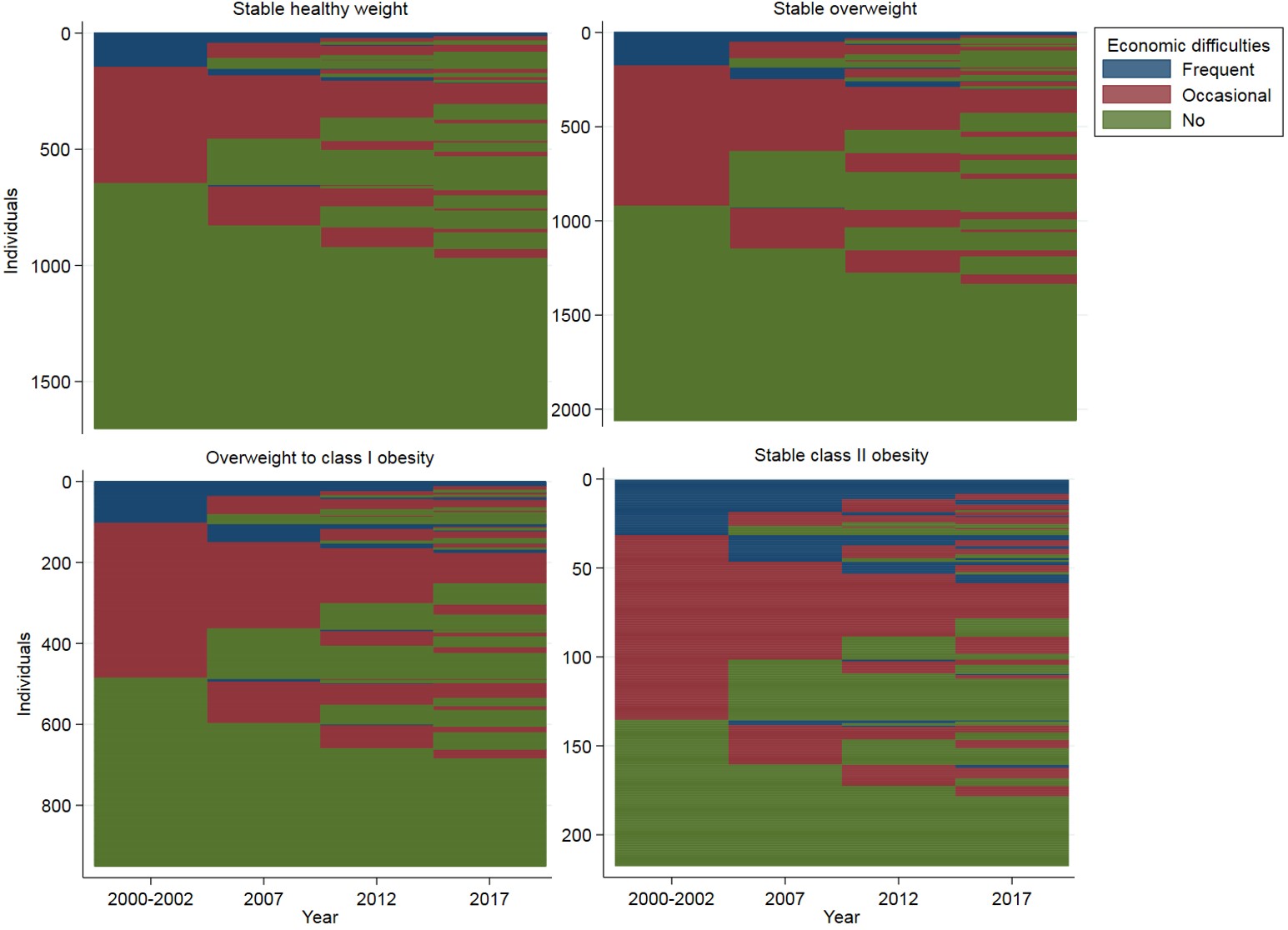

Supplement: Supplementary file 1 [file ijerph-17-03668-s001.zip › Figure3_IJERPH.jpg]
